# Expanded PCR Panel Testing for Identification of Respiratory Pathogens and Coinfections in Influenza-like Illness

**DOI:** 10.3390/diagnostics13122014

**Published:** 2023-06-09

**Authors:** Pallavi Upadhyay, Jairus Reddy, Teddie Proctor, Oceane Sorel, Harita Veereshlingam, Manoj Gandhi, Xuemei Wang, Vijay Singh

**Affiliations:** 1 HealthTrackRx R&D Division, Denton, TX 76207, USA; pallavi.upadhyay@healthtrackrx.com (P.U.); jay.reddy@healthtrackrx.com (J.R.); 2 Thermo Fisher Scientific, 180 Oyster Point Blvd, South San Francisco, CA 94080, USA; teddie.proctor@thermofisher.com (T.P.); oceane.sorel@thermofisher.com (O.S.); harita.veereshlingam@thermofisher.com (H.V.); xuemei.wang@thermofisher.com (X.W.)

**Keywords:** respiratory tract infections, COVID-19, influenza-like illness, multiplex PCR, syndromic panels

## Abstract

While COVID-19 has dominated Influenza-like illness (ILI) over the past few years, there are many other pathogens responsible for ILI. It is not uncommon to have coinfections with multiple pathogens in patients with ILI. The goal of this study was to identify the different organisms in symptomatic patients presenting with ILI using two different high throughput multiplex real time PCR platforms. Specimens were collected from 381 subjects presenting with ILI symptoms. All samples (nasal and nasopharyngeal swabs) were simultaneously tested on two expanded panel PCR platforms: Applied Biosystems™ TrueMark™ Respiratory Panel 2.0, OpenArray™ plate (OA) (32 viral and bacterial targets); and Applied Biosystems™ TrueMark™ Respiratory Panel 2.0, TaqMan™ Array card (TAC) (41 viral, fungal, and bacterial targets). Results were analyzed for concordance between the platforms and for identification of organisms responsible for the clinical presentation including possible coinfections. Very good agreement was observed between the two PCR platforms with 100% agreement for 12 viral and 3 bacterial pathogens. Of 381 specimens, approximately 58% of the samples showed the presence of at least one organism with an important incidence of co-infections (~36–40% of positive samples tested positive for two and more organisms). *S. aureus* was the most prevalent detected pathogen (~30%) followed by SARS-CoV-2 (~25%), Rhinovirus (~15%) and HHV6 (~10%). Co-infections between viruses and bacteria were the most common (~69%), followed by viral-viral (~23%) and bacterial-bacterial (~7%) co-infections. These results showed that coinfections are common in RTIs suggesting that syndromic panel based multiplex PCR tests could enable the identification of pathogens contributing to coinfections, help guide patient management thereby improving clinical outcomes and supporting antimicrobial stewardship.

## 1. Introduction

Respiratory tract infections (RTIs) are one of the major public health concerns across the world. As opposed to the previous belief that RTIs are caused by a single pathogen, studies have shown that most RTIs are a result of a combination of bacterial and/or viral pathogens coinfecting the host, leading to increased disease severity [1]. While respiratory viruses such as influenza A/B, respiratory syncytial virus (RSV), human metapneumovirus (hMPV), rhinoviruses (HRV), etc. dominate the RTIs, 10–15% of patients develop secondary bacterial coinfection [2]. Due to their high transmission rate, RTIs are accountable for significant mortality and hospitalizations, thus posing a significant healthcare and economic burden globally. According to the WHO, lower RTIs are the fourth leading cause of death worldwide [3]. Among them, RTIs caused by RSV are one of the major causes of hospitalization each year [4,5]. In developing countries, RTIs are the most common cause of mortality in children below five years of age, and account for 20–40% of the total volume of primary care visits in the pediatric population [6]. Prior to the COVID-19 pandemic, seasonal influenza represented the highest burden in terms of both incidence and cost among all preventable diseases [7,8,9]. According to a study, in 2015, seasonal influenza in the United States resulted in an estimated economic burden of $11.2 billion, of which, $8.0 billion were accounted toward indirect costs [10]. It is worth noting that the annual economic burden of non-influenza viral RTIs accounted for a much higher cost of $40 billion [11]. The COVID-19 pandemic added another layer of economic burden, with hospitalizations increasing in 2020 and 2021, along with preventative measures including prolonged quarantines and isolation [12,13]. The estimated burden of COVID-19 alone in the United Kingdom was 39.6 billion pounds, and when accounting for mitigation strategies (including quarantine) the direct health-related burden increased to 53.1 billion pounds [13]. In the United States, between March 2020 and February 2021, the loss of work hours alone represented an estimated cost of $138 billion among full-time workers [14].

The clinical presentations of RTIs have overlapping symptoms, often showing influenza-like illness (ILI). While COVID-19 has dominated ILI over the past two years, other pathogens can potentially be responsible for ILI. In addition, it is not uncommon to detect coinfections with multiple pathogens in patients presenting with ILI [15,16,17]. Therefore, insights into coinfecting pathogens could help with accurate disease prognosis, patient care management, and outcomes. In the past two decades, advancement in the nucleic acid amplification test (NAAT)-based techniques, especially multiplex PCR, has revolutionized the field of infectious disease diagnostics. The use of a syndromic panel-based testing approach in RTIs has been shown to improve patient outcomes in a timely manner [18]. The goal of this study was to identify the various respiratory pathogens in symptomatic patients presenting with ILI/RTI using two different high-throughput multiplex real-time PCR platforms.

## 2. Material and Methods

### 2.1. Specimen Collection

Nasopharyngeal and anterior nasal swabs were both collected from 381 individuals of all ages who exhibited symptoms of an acute respiratory tract infection that developed over the past seven days. The specimens were collected between February 2021 and May 2021, from three geographically diverse locations in the United States. Eligibility was determined using the following inclusion symptoms criteria: ILI symptoms (fever, chills, runny or stuffy nose, body aches, headache, and/or feeling excessively tired), a new loss of taste or smell, a new or worsening cough or sore throat, shortness of breath, difficulty breathing, nausea, vomiting, or diarrhea. Symptoms were further classified as systemic or respiratory symptoms for analysis (Table 1). Individuals who had received a COVID-19 vaccine or any antiviral therapy were not eligible to participate. Nasopharyngeal and anterior nasal swabs were collected in accordance with the instructions in the appropriate collection kit package insert and placed into the appropriately labeled containing collection medium. Deidentified samples were stored at a temperature between 2 °C and 8 °C and shipped to the testing sites. All protocols have been approved by the author’s Institutional Review Board for human subjects (IRB approval number: PCP0068620).

### 2.2. Multiplex Single Panel Testing

All samples were tested using the TaqPath™ COVID-19, FluA, FluB Combo Kit (Thermo Fisher Scientific, Waltham, MA, USA), the Lyra^®^ Influenza A+B Assay (Quidel, San Diego, CA, USA), and the Lyra^®^ SARS-CoV-2 Assay (Quidel, San Diego, CA, USA) according to the manufacturer’s instructions for use. Sample extraction was performed using both the KingFisher™ Flex Purification System (Thermo Fisher Scientific) and the NUCLISENS^®^ EASYMAG^®^ (BioMérieux, Durham, NC, USA). After extraction, all real-time PCR was performed using an Applied Biosystems™ 7500 Fast Dx instrument for both assays. After initial testing, remnant specimens were stored at −80 °C.

### 2.3. Multiplex Expanded Panel Testing

Remnant samples (nasal and nasopharyngeal swabs) were simultaneously tested on two expanded panel PCR platforms: the Applied Biosystems™ TrueMark™ Respiratory Panel 2.0, TaqMan™ Array card (TAC) (41 viral, fungal, and bacterial targets) and the Applied Biosystems™ TrueMark™ Respiratory Panel 2.0, OpenArray™ plate (OA) (32 viral and bacterial targets) at the HealthTrackRx Laboratory located in Denton, TX, USA. TAC cards were prepared and loaded according to the manufacturers’ instructions. The OA panels were run according to manufacturer instructions as previously described [19]. Both TAC and OA panels were run on the QuantStudio12K flex platform. PCR cycling was performed using the following program: single cycle of enzyme activation (95 °C) for 10 min, followed by 40 cycles of denaturation (95 °C) for 15 s, and annealing/extension (60 °C). Results were analyzed for concordance between the two platforms for different organisms responsible for the clinical presentation, as well as for coinfections.

## 3. Results

A total of 381 deidentified respiratory samples (nasal and nasopharyngeal swabs) were collected from individuals experiencing ILI symptoms. SARS-CoV-2 was detected in only 84 samples (22%), and all the samples were negative for Flu A or Flu B with either of the two influenza detection assays (Table 2). These data suggested that the remaining 297 individuals (78%) that presented with ILI and tested negative for SARS-CoV-2, Flu A, and Flu B, might be infected with other respiratory pathogens.

**Figure 1:**
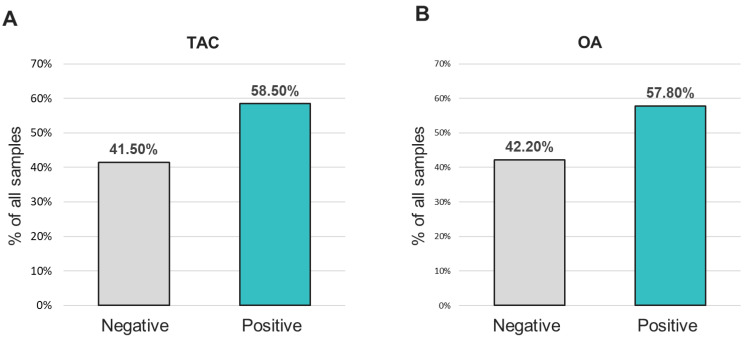
Positivity rates for any organism from samples tested with the (**A**) TAC or (**B**) OA platform.

**Figure 2:**
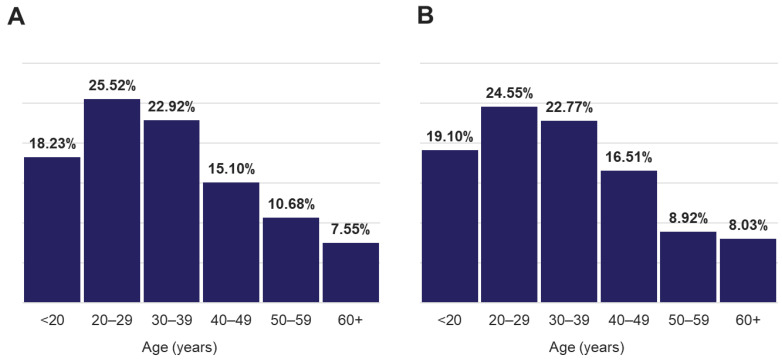
Age distribution of (**A**) all individuals and (**B**) that were tested positive for any target.

**Figure 3:**
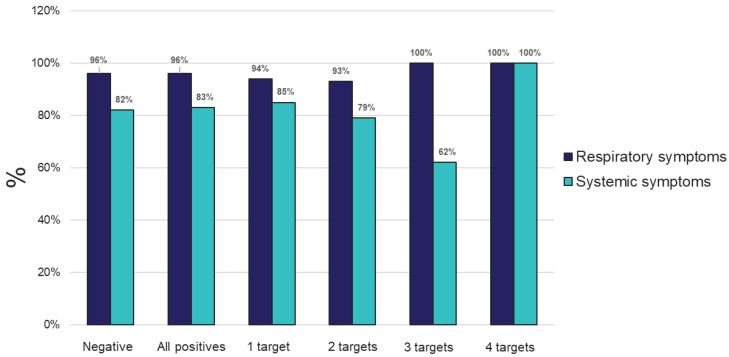
Percent of individuals experiencing respiratory or systemic symptoms that tested negative, positive for any (All positives), or for 1, 2, 3, or 4 targets from samples tested using the TAC platform.

To identify other respiratory pathogens that could be responsible for the ILI symptoms, the entire sample cohort was tested on two expanded panel PCR platforms (TAC and OA) for the presence of various viral, bacterial and fungal respiratory pathogens. Of 381 specimens, approximately 58% of the samples showed the presence of at least one organism (TAC: 223/381 (58.5%); OA 218/381 (57.8%)) (Figure 1 and Table S1). The distribution of all positive specimens by age range was similar to that of the total population, and revealed more samples collected from the 20–29 and 30–39 age groups, which comprised approximately 47% of the total positive cases (Figure 2). Interestingly, both positive and negative cohorts exhibited similar respiratory and systemic symptoms (Figure 3).

The analysis demonstrated very good concordance between the two PCR platforms (TAC and OA). The data showed 100% agreement (PPA, NPA) for the 12 viral pathogens: SARS-CoV-2, Coronaviruses (NL63, OC43, 229E), Parainfluenza virus (HPIV) 2, HPIV 3, Pan-enterovirus, Enterovirus D68, Respiratory syncytial virus B (RSV-B), HHV4-EBV, HHV5-CMV, and HHV6; and three bacterial pathogens: *Hemophilus influenzae*, *Klebsiella pneumoniae* and *Streptococcus pneumoniae* (Table 3). Regarding the other detected pathogens, *Staphylococcus aureus* (98.97% PPA, 100% NPA) and hRV (100% PPA, 99.10% NPA) were observed (Table 4). One sample tested positive for hMPV on TAC but did not test positive for this organism on OA (Table S2). In addition, *Moraxella catarrhalis* and *Pneumocystis jirovecii* were detected positive on TAC (18 and 2 samples, respectively). Since TaqMan assays for *Moraxella catarrhalis* and *Pneumocystis jirovecii* were not available on OA, an agreement could not be established for these two pathogens (Table S2).

**Figure 4:**
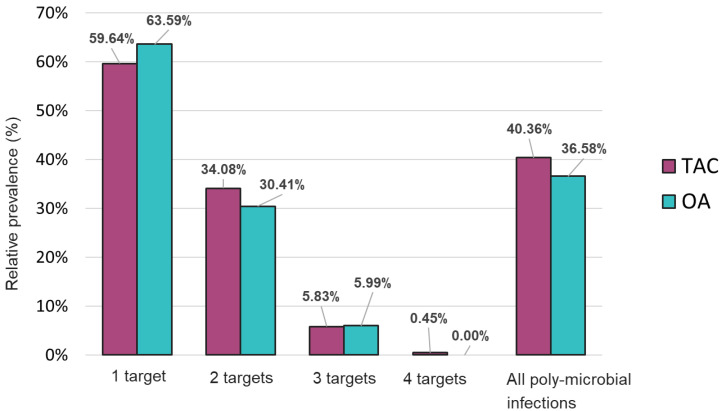
Distribution of mono- and poly-microbial infections from samples tested with the TAC or OA platform among all positive samples.

Comparable results were also obtained on both platforms regarding detection of mono- and poly-microbial infections (Figure 3). Mono-microbial infections were the most prevalent among positive samples, with 59.64% and 63.59% on TAC and OA, respectively (Figure 4 and Table S2). However, data collected using both platforms also showed an important incidence of coinfections, with 40.36% and 36.58% of positive samples testing positive for two or more organisms on TAC and OA, respectively (Figure 4). Among all coinfections, two pathogens were detected in almost one third of the positive cases (34.08% on TAC, 30.41% on OA) (Figure 3 and Table S4), and three pathogens were detected in around 6% of all positive cases (5.83% on TAC, 5.99% on OA) (Figure 3 and Table S5). Finally, one sample tested positive for four organisms on TAC, while on OA a maximum of three co-infecting organisms were detected (Figure 4 and Figure S6). The few discrepancies in the distribution of mono- and poly-microbial infections between the two platforms can be mostly explained by the fact that detection of *M. catarrhalis* and *P. jirovecii* is not included on OA.

**Figure 5:**
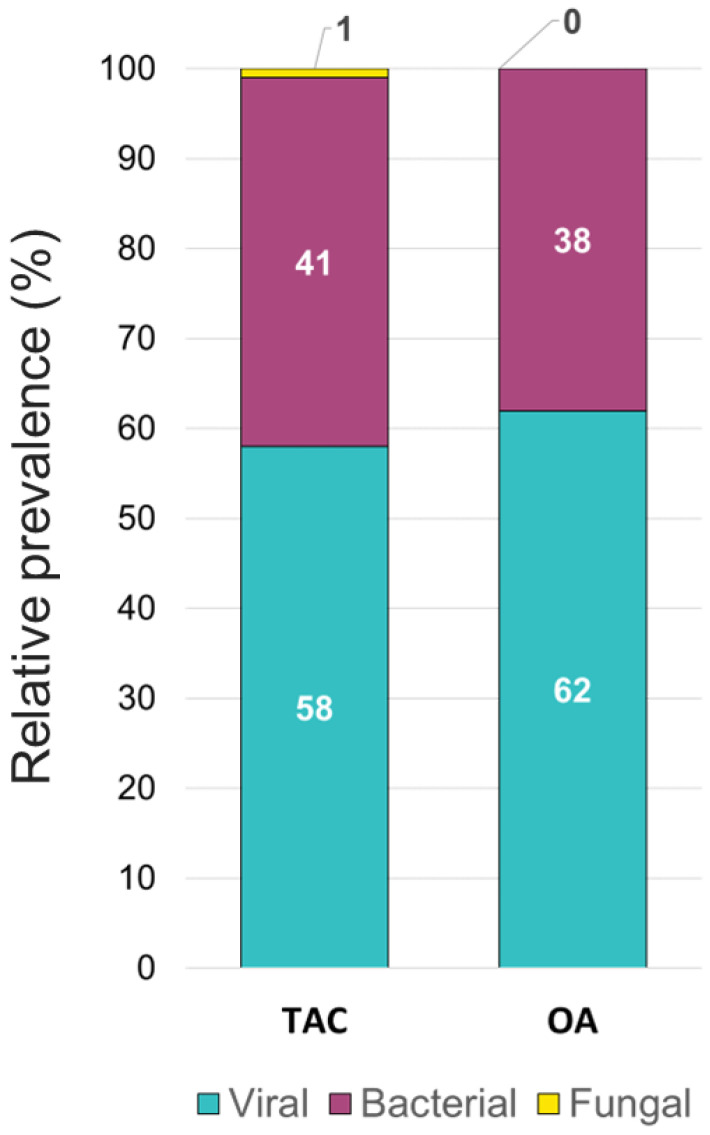
Relative prevalence of types of pathogens among all positive specimens identified using the TAC and the OA platform.

**Figure 6:**
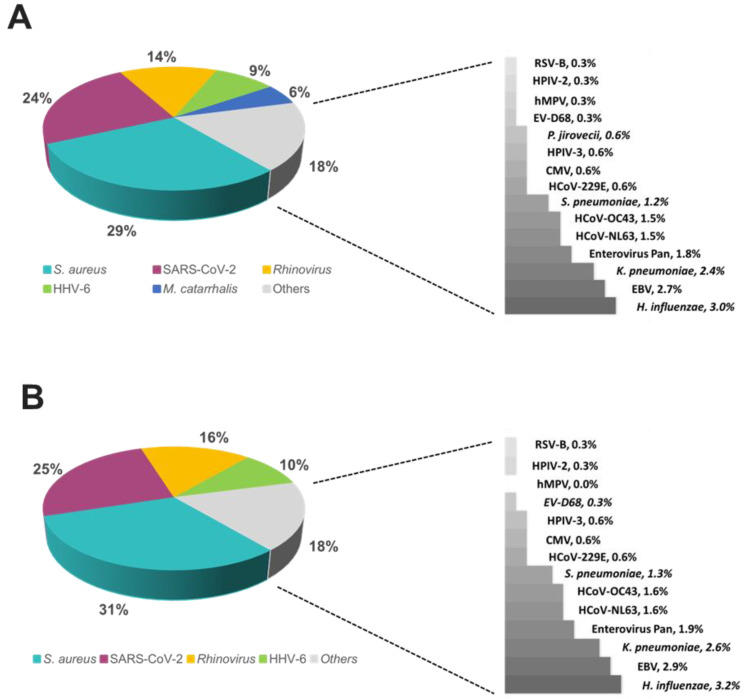
Relative prevalence of organisms identified using the (**A**) TAC or (**B**) OA platform among all positive specimens.

**Figure 7:**
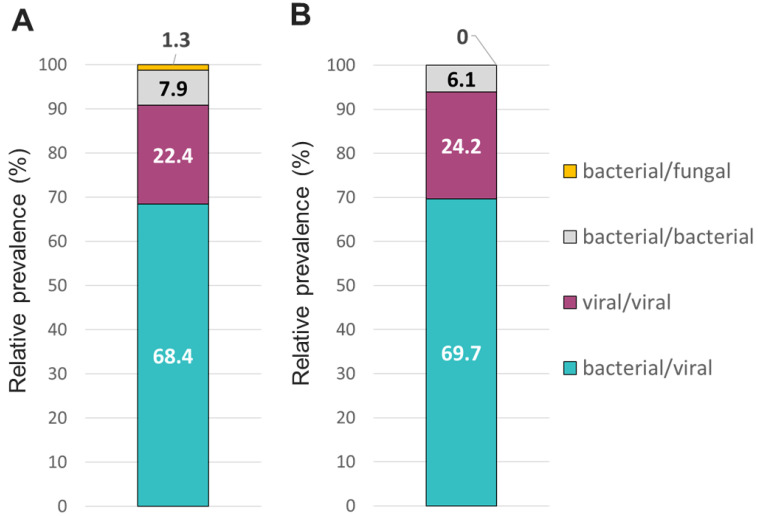
Relative prevalence of coinfecting types of pathogens using the (**A**) TAC or (**B**) OA platform among specimens that tested positive for two or more pathogens.

**Figure 8:**
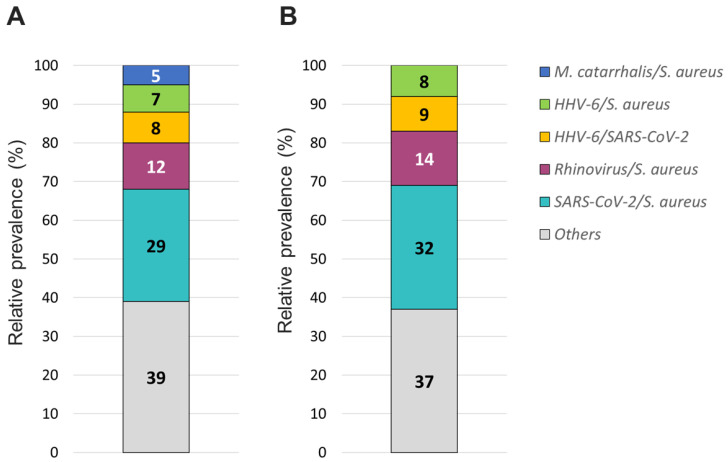
Relative prevalence of coinfecting organisms using the (**A**) TAC or (**B**) OA platform among collected specimens that tested positive for two organisms.

The overall relative prevalence of the type of pathogens among all positive specimens was analyzed and showed that viral infections (~60%) were the most prevalent followed by bacterial infections (~40%) while fungal infection was rarely detected (1% on TAC) (Figure 5). A detailed analysis of the detected pathogens showed that *S. aureus* was the most prevalent on both platforms (~30%) followed by SARS-CoV-2 (~25%), HRV (~15%) and HHV-6 (~10%) (Figure 6). *M. catarrhalis* was also detected in 6% of the positive samples on TAC (Figure 6A) which is not included on OA (Figure 6B). The remaining organisms accounted for around 18% of all infections detected on both platforms and comprised pathogens that had lower relative prevalence (Figure 6). Coinfections between viruses and bacteria were the most common (~69%), followed by viral–viral (~23%) and bacterial–bacterial (~7%) coinfections (Figure 7). Coinfections between bacteria and fungi were rare (1.3% of all coinfections) (Figure 7) and detected only on TAC. *S. aureus* was the most prevalent bacterial coinfecting pathogen, where SARS-CoV-2/*S. aureus* coinfections were the most common (~30%), followed by HRV/*S. aureus* (~13%), HHV6/*S. aureus* (~8%), and *M. catarrhalis*/*S. aureus* (~5%) (Figure 8). In addition, around 9% of all coinfections involved SARS-CoV-2/HHV6 (Figure 8).

## 4. Discussion

All patients enrolled in this study displayed symptoms of ILI, and in light of the fact that these patients were presenting to the hospital during the COVID-19 pandemic, these samples were tested using a multiplex PCR panel for the presence of SARS-CoV-2, Flu A, and Flu B. While a proportion of samples were positive for SARS-CoV-2, none of the samples were positive for influenza. While public health experts had predicted a “twindemic” in the winter of 2021, the complete absence of influenza was a little surprising. In hindsight, strict economic lockdowns and restrictions on international travel, along with other non-pharmaceutical interventions enacted to curtail the spread of COVID-19, can explain the historically low influenza activity in the United States during 2020 and 2021 (FluSurv-NET) [20]. It was surprising, however, that only around 20% of the ILI patient samples tested positive for the presence of SARS-CoV-2. This indicated that patients presenting with symptoms of ILI had infections by respiratory pathogens other than SARS-CoV-2 and influenza.

To study this possibility, we evaluated these samples using an expanded respiratory pathogen PCR panel. We were able to detect the presence of at least one ILI-causing pathogen in around 57% of the samples. We also report significant coinfections, both bacterial and viral, which can potentially complicate the disease manifestation and can impact the treatment of these patients. Importantly, rhinovirus was detected in 23.8% of the patient samples. In pediatric patients, respiratory viral burden was found to be significantly higher in hospitalized patients as opposed to the outpatient population [21]. The same study demonstrated that rhinovirus was a significant cause of bronchiolitis and pneumonia [21]. In addition, some viral infections can also predispose to bacterial superinfections, and the use of expanded panels could be valuable to detect such complications. Multiple studies have also shown the advantage of utilizing expanded PCR panels in diagnosing the cause of respiratory infections over the use of smaller PCR panels targeting one or two pathogens such as influenza and RSV [22,23,24]. In addition, studies have shown that that multiplex PCR panels can reduce the use of antimicrobials and the length of hospital stay associated with respiratory infections as compared to conventional diagnostic methods [25,26].

The data presented in this study pertain to an outpatient population wherein the policies for using NAAT-based technology for the detection of respiratory pathogens presents some ambiguity. The current Infectious Disease Society of America (IDSA) and American Thoracic Society (ATS) guidelines for detecting and treating community acquired pneumonia still recommend empiric therapy for the treatment of bacterial pneumonia. Although the advantage of PCR in providing quick results is acknowledged, its use over the classical diagnostic methods (sputum and blood cultures) has not been recommended due to the lack of data regarding the clinical advantage of the technique [27]. In addition, according to Centers of Medicare and Medicaid policy dictating the reimbursement of laboratory tests, the use of expanded PCR panels (>5 pathogens) in an outpatient setting is not considered medically necessary [28]. Although ILI are usually self-limited conditions, some may result in complications such as lower respiratory tract infections, including pneumonia, particularly in high-risk populations [29,30]. Although our dataset comprised a small cohort of patients aged 60 years and older, these high-risk patients showed respiratory infections with a significant number of clinically relevant organisms, including polymicrobial infections. The presence of these organisms can lead to hospitalization and affect treatment options and disease prognosis especially if not treated promptly with appropriate antimicrobials. For instance, the presence of *S. aureus* was found in almost 30% of patient samples. Although *S. aureus* could be part of the commensal flora in healthy carriers, it can also cause invasive illnesses, including pneumonia [31]. Among all *S. aureus* strains, Methicillin-resistant *Staphylococcus aureus* (MRSA) are a major threat for public health. A multi-center prospective study of adult patients hospitalized with community-acquired pneumonia (CAP) showed that a significant number of hospitalized patients with similar overlapping symptoms were treated with anti-MRSA antibiotics [32]. Current IDSA and ATS guidelines recommend against empiric antibiotic treatment of MRSA, but addition of vancomycin and linezolid is recommended if MRSA-associated CAP is suspected [33]. It has been suggested that rapid detection of *S. aureus* and other bacterial causative agents of CAP would result in an overall reduction in the use of anti-MRSA antibiotics, allowing for better antibiotic stewardship [32]. Importantly, a recent study suggested that utilization of multiplex bacterial PCR may reduce inappropriate antibiotic use, potentially promoting good antibiotic stewardship in hospitalized patients with bacterial pneumonia [34]. In the context of antimicrobial stewardship, early detection of infecting pathogens in patients presenting ILI symptoms can also help discriminate between viral, bacterial, and fungal infections, and help determine the appropriate treatment decision. Finally, in this study, over 40% of tested specimens did not test positive for any pathogens despite experiencing ILI symptoms. These patients might be infected with pathogens that were not included in the expanded panel, or they might experience non-infectious diseases such as allergies for whom antibiotic treatment is not appropriate. This finding further emphasizes the potential role that expanded PCR panels could play in guiding antimicrobial decisions.

Another practical aspect of this study was the assessment of two different PCR technologies for the expanded panel testing. It has been previously demonstrated that both TAC [35,36] and OA [2,19] platforms can be employed for the simultaneous detection of multiple respiratory pathogens. Both platforms utilize the TaqMan^®^ probe-based multiplexed, real-time PCR technology. We observed 100% concordance for most of the detected pathogens on both platforms, which showed that our data are consistent despite using two different testing methods. These findings further highlight the utility of a flexible high-throughput real-time PCR system such as QuantStudio12K Flex, which can be equipped with heating blocks to run either platform depending on the sample volume burden of the testing laboratory, helping maintain a quick turnaround time for results which can be crucial to patient care.

One of the limitations of this study is the lack of data regarding patient outcomes, which does not allow us to conclude on the impact of utilizing a large respiratory panel PCR on patient care and management. Moreover, this study contained a very limited subset of patients aged >60 years that are at higher risk of ILI complications and hospitalization. Future studies should focus on high-risk populations including detailed follow up data to assess how expanded multiplex PCR panels could improve clinical outcomes.

This study represents a significant advancement in our understanding of the diagnosis of ILI-causing pathogens in an outpatient population where the diagnostic guidelines are not as clear as in the case of hospitalized patients. The data presented clearly delineates the superiority of a multiplexed PCR approach in detecting respiratory bacterial, viral, and fungal pathogens over tests that detect single pathogens. This is especially true in the backdrop of a global pandemic, where the focus and availability of diagnostic testing is restricted. Thus, in the context of ILI complications, our data highlight the potential role of using expanded RTI panels to adapt treatment decisions and improve patient outcomes, especially in the population that is at highest risk of complications of ILI.

## Figures and Tables

**Table 1:** Classification of symptoms.

Systemic Symptoms	Respiratory Symptoms
Fever	Runny nose
Chills	Cough
Body aches	Sore throat
Headache	Shortness of breath
Fatigue	
Loss of taste or smell	
Nausea	
Vomiting	
Diarrhea	

**Table 2:** Performance comparison of TaqPath™ COVID-19, FluA, FluB Combo Kit, Lyra^®^ SARS-CoV-2 Assay and Lyra^®^ Influenza A+B RT-PCR Assay for detection of SARS-CoV-2, Flu A and Flu B. * 1 sample was tested positive on TaqPath™ COVID-19, FluA, FluB Combo Kit and invalid for SARS-CoV-2 detection on Lyra^®^ SARS-CoV-2 Assay). N/A: PPA not applicable due to a lack of positive samples.

		Lyra^®^ SARS-CoV-2 Assay			Lyra^®^ Influenza A+B RT-PCR Assay					
		SARS-CoV-2			Flu A			Flu B		
		Positive	Negative	Total	Positive	Negative	Total	Positive	Negative	Total
TaqPath™ COVID-19, FluA, FluB Combo Kit	Positive	76	7	84 *	0	0	0	0	0	0
	Negative	3	294	297	0	381	381	0	381	381
	Total	79	301	381	0	381	381	0	381	381
PPA (95% CI)		96.2% (89.3% to 99.2%)			N/A (0.0% to 100.0%)			N/A (0.0% to 100.0%)		
NPA (95% CI)		97.7% (95.3% to 99.1%)			100.0% (99.0% to 100.0%)			100.0% (99.0% to 100.0%)		

**Table 3:** Organisms that showed 100% agreement statistics between the Applied Biosystems™ TrueMark™ Respiratory Panel 2.0, TaqMan™ Array card (TAC) and the Applied Biosystems™ TrueMark™ Respiratory Panel 2.0, OpenArray™ plate (OA).

Organism Name (Number of Specimens)		
*Haemophilus influenzae * (*N* = 10)	HHV-6 (*N* = 30)	Coronavirus NL63 (*N* = 5)
*Enterovirus D68* (*N* = 1)	RSV-B (*N* = 1)	Coronavirus OC43 (*N* = 5)
*Klebsiella pneumoniae * (*N* = 8)	EBV (*N* = 9)	Coronavirus 229E (*N* = 2)
*Streptococcus pneumoniae * (*N* = 4)	CMV (*N* = 2)	HPIV-2 (*N* = 1)
Pan-enterovirus (*N* = 6)	SARS-CoV-2 (*N* = 78)	HPIV-3 (*N* = 2)
Overall agreement (TAC vs. OA)		100.00%

**Table 4:** Overall agreement statistics between the Applied Biosystems™ TrueMark™ Respiratory Panel 2.0, TaqMan™ Array card (TAC) and the Applied Biosystems™ TrueMark™ Respiratory Panel 2.0, OpenArray™ plate (OA) for detection of Staphylococcus aureus and Rhinovirus.

	TAC				
**OA**	***Staphylococcus aureus***		**Positive**	**Negative**	**Total**
		**Positive**	96	0	96
		**Negative**	1	284	285
		**Total**	97	284	381
	**Positive Percent Agreement ** [95% CI]		**98.97% ** [94.39%–99.82%]		
	**Negative Percent Agreement ** [95% CI]		**100.00% ** [98.67%–100.00%]		
	***Rhinovirus***		**Positive**	**Negative**	**Total**
		**Positive**	47	3	50
		**Negative**	0	331	331
		**Total**	47	334	381
	**Positive Percent Agreement ** [95% CI]		**100.00% ** [92.44%–100.00%]		
	**Negative Percent Agreement ** [95% CI]		**99.10% ** [97.39%–99.69%]		

## Data Availability

The data presented in this study are available on request from the corresponding author. The data are not publicly available due to restrictions on public data sharing as laid out in the IRB approval statement.

## Supplementary Materials

The following supporting information can be downloaded at: https://www.mdpi.com/article/10.3390/diagnostics13122014/s1.
