# Supplementary material for: Expanded PCR Panel Testing for Identification of Respiratory Pathogens and Coinfections in Influenza-like Illness
Source: Diagnostics (Basel). 2023 Jun 9;13(12):2014. doi: 10.3390/diagnostics13122014 (PMC10297358; doi:10.3390/diagnostics13122014)
Supplement: Supplementary file 1 [file diagnostics-13-02014-s001.zip › diagnostics-2428177-supplementary.pdf]

Supplemental Tables

	TAC		OA	
Number of positive targets	# of positive samples	% of total samples	# of positive samples	% of total samples
0	158	41.47%	164	43.04%
1	133	34.91%	138	36.22%
2	76	19.95%	66	17.32%
3	13	3.41%	13	3.41%
4	1	0.26%	0	0%
Total	381	100.00%	381	100.00%

Table S1: Number of positive samples and positivity rate for one, two, three or four organisms from samples tested with the TAC platform.

	TAC		OA	
Positive targets	# of positive samples	Relative prevalence (%) among all positives	# of positive samples	Relative prevalence (%) among all positives
Staphylococcus aureus	96	29.3%	97	31.2%
SARS-CoV-2	78	23.8%	78	25.1%
Rhinovirus	47	14.3%	50	16.1%
Human Herpesvirus 6	30	9.1%	30	9.6%
Moraxella catarrhalis	18	5.5%	N/A	N/A
Haemophilus influenzae	10	3.0%	10	3.2%
Human Herpesvirus 4-EBV	9	2.7%	9	2.9%
Klebsiella pneumoniae	8	2.4%	8	2.6%
Enterovirus Pan	6	1.8%	6	1.9%
Coronavirus NL63	5	1.5%	5	1.6%
Coronavirus OC43	5	1.5%	5	1.6%
Streptococcus pneumoniae	4	1.2%	4	1.3%
Coronavirus 229E	2	0.6%	2	0.6%
Human Herpesvirus 5-CMV	2	0.6%	2	0.6%
Parainfluenza virus 3	2	0.6%	2	0.6%
Pneumocystis jirovecii	2	0.6%	N/A	N/A
Enterovirus D68	1	0.3%	1	0.3%
Metapneumovirus (hMPV)	1	0.3%	0	0.0%
Parainfluenza virus 2	1	0.3%	1	0.3%
Respiratory Syncytial Virus B	1	0.3%	1	0.3%

Table S2: Number of positive samples and relative prevalence of organisms identified using the TAC or OA platform among all positive specimens. (N/A : Not applicable because *Moraxella catarrhalis* and *Pneumocystis jirovecii* were included on TAC but not tested on OA)

	TAC		OA	
Positive targets	# of positive samples	Relative prevalence (%) among specimen positive for 1 target	# of positive samples	Relative prevalence (%) among specimen positive for 1 target
Staphylococcus aureus	41	31%	47	34%
SARS-CoV-2	38	29%	40	29%
Rhinovirus	24	18%	25	18%
Human Herpesvirus 6	9	7%	9	7%
Moraxella catarrhalis	5	4%	N/A	N/A
Coronavirus NL63	4	3%	4	3%
Coronavirus 229E	2	2%	2	1%
Coronavirus OC43	2	2%	2	1%
Human Herpesevirus 4-EBV	2	2%	2	1%
Klebsiella pneumoniae	2	2%	3	2%
Haemophilus influenzae	1	1%	1	1%
Metapneumovirus (hMPV)	1	1%	0	0%
Respiratory Syncytial Virus B	1	1%	1	1%
Streptococcus pneumoniae	1	1%	1	1%
**Total**	**133**	**100%**	**137**	**100%**

Table S3: Number of positive samples and relative prevalence of organisms identified using the TAC or OA platform among specimens that tested positive for only 1 target. (N/A : Not applicable because *Moraxella catarrhalis* was included on TAC but not tested on OA)

	TAC		OA	
Positive targets	# of positive samples	Relative prevalence (%) among specimen positive for 2 targets	# of positive samples	Relative prevalence (%) among specimen positive for 2 targets
SARS-CoV-2 / S. aureus	22	29%	21	32%
Rhinovirus/Staphylococcus aureus	9	12%	9	14%
Human Herpesvirus 6/SARS-CoV-2	6	8%	6	9%
Human Herpesvirus 6/Staphylococcus aureus	5	7%	5	8%
Moraxella catarrhalis/Staphylococcus aureus	4	5%	ND	ND
Enterovirus Pan/Rhinovirus	3	4%	3	5%
Human Herpesvirus 6/Rhinovirus	3	4%	3	5%
Moraxella catarrhalis/SARS-CoV-2	3	4%	N/A	N/A
Haemophilus influenzae/Staphylococcus aureus	2	3%	2	3%
Klebsiella pneumoniae/Staphylococcus aureus	2	3%	2	3%
Parainfluenza virus 3/Staphylococcus aureus	2	3%	2	3%
Coronavirus OC43/Haemophilus influenzae	1	1%	1	2%
Coronavirus OC43/Klebsiella pneumoniae	1	1%	1	2%
Coronavirus OC43/Staphylococcus aureus	1	1%	1	2%
Haemophilus influenzae/Human Herpesvirus 6	1	1%	1	2%
Haemophilus influenzae/Rhinovirus	1	1%	1	2%
Haemophilus influenzae/SARS-CoV-2	1	1%	2	3%
Haemophilus influenzae/Streptococcus pneumoniae	1	1%	1	2%
Human Herpesvirus 4(EBV)/Klebsiella pneumoniae	1	1%	1	2%
Human Herpesvirus 4 (EBV)/SARS-CoV-2	1	1%	1	2%
Human Herpesvirus 5 (CMV)/Klebsiella pneumoniae	1	1%	ND	ND
Klebsiella pneumoniae/Moraxella catarrhalis	1	1%	N/A	N/A
Moraxella catarrhalis/Parainfluenza virus 2	1	1%	N/A	N/A
Moraxella catarrhalis/Rhinovirus	1	1%	N/A	N/A
Pneumocystis jirovecii/Staphylococcus aureus	1	1%	N/A	N/A
Rhinovirus/SARS-CoV-2	1	1%	3	5%
**Total**	**76**	**100%**	**66**	**100%**

Table S4: Number of positive samples and relative prevalence of organisms identified using the TAC or OA platform among specimens that tested positive for 2 targets. (ND: Not detected positive for both targets; N/A : Not applicable because *Moraxella catarrhalis* and *Pneumocystis jirovecii* were included on TAC but not tested on OA)

Sample ID	Target 1	Target 2	Target 3	All 3 targets detected in TAC	All 3 targets detected in OA
1-302-194	Enterovirus D68	Enterovirus Pan	Rhinovirus	Yes	Yes
1-302-017	Enterovirus Pan	Human Herpesvirus 4 (EBV)	Rhinovirus	Yes	Yes
1-302-160	Human Herpesvirus 4 (EBV)	Human Herpesvirus 6	SARS-CoV-2	Yes	Yes
1-305-009	Human Herpesvirus 5 (CMV)	Human Herpesvirus 6	Staphylococcus aureus	Yes	Yes
1-302-176	Human Herpesvirus 6	Rhinovirus	Staphylococcus aureus	Yes	Yes
1-302-184	Coronavirus NL63	SARS-CoV-2	Staphylococcus aureus	Yes	Yes
1-302-133	Human Herpesvirus 4 (EBV)	SARS-CoV-2	Staphylococcus aureus	Yes	Yes
1-305-080	Human Herpesvirus 6	SARS-CoV-2	Staphylococcus aureus	Yes	Yes
1-302-098	Haemophilus influenzae	Human Herpesvirus 4 (EBV)	Streptococcus pneumoniae	Yes	Yes
1-302-143	Enterovirus Pan	Rhinovirus	Streptococcus pneumoniae	Yes	Yes
1-302-242	Human Herpesvirus 6	Moraxella catarrhalis	Staphylococcus aureus	Yes	N/A
1-304-013	Haemophilus influenzae	Pneumocystis jirovecii	SARS-CoV-2	Yes	N/A
1-302-205	Moraxella catarrhalis	Rhinovirus	SARS-CoV-2	Yes	N/A
1-305-019	Human Herpesvirus 5 (CMV)	Klebsiella pneumoniae	Rhinovirus	No	Yes
1-302-167	Rhinovirus	SARS-CoV-2	Staphylococcus aureus	No	Yes
1-302-109	Human Herpesvirus 4-EBV	Human Herpesvirus 6	Staphylococcus aureus	Yes but 4 total targets were detected	Yes
**Total**				**13**	**13**

Table S5: Types of organisms identified using the TAC or OA platform among specimens that tested positive for 3 targets. (N/A : Not applicable because *Moraxella catarrhalis* and *Pneumocystis jirovecii* were included on TAC but not tested on OA)

Sample ID	Target 1	Target 2	Target 3	Target 4
1-302-109	Human Herpesvirus 4 (EBV)	Human Herpesvirus 6	Moxarella catarrhalis	Staphylococcus aureus

Table S6: Types of organisms identified using the TAC platform in the specimen that tested positive for 4 targets.

Sample ID	Target 1	Target 2	Target 3
1-302-167	Rhinovirus	SARS-CoV-2	
1-305-019	Rhinovirus	***Klebsiella pneumoniae***	CMV
1-302-213	Rhinovirus	***Staphylococcus aureus***	
1-305-057	Rhinovirus	***Staphylococcus aureus***	
1-305-069	EBV	***Klebsiella pneumoniae***	
1-302-137	***Haemophilus influenzae***	Rhinovirus	
1-302-139	Rhinovirus		
1-305-040	EBV	SARS-CoV-2	
1-305-010	SARS-CoV-2		
1-305-034	EBV		
1-305-100	Human Herpesvirus 6		
1-301-008	***Staphylococcus aureus***		
1-305-037	***Staphylococcus aureus***		
1-305-047	***Staphylococcus aureus***		
1-305-083	***Staphylococcus aureus***		
1-305-086	***Staphylococcus aureus***		
1-308-003	***Staphylococcus aureus***		
1-302-069	**NEGATIVE**		
1-302-135			
1-302-207			
1-302-232			
1-305-002			
1-305-007			
1-305-017			
1-305-018			
1-305-022			
1-305-027			
1-305-043			
1-305-099			

Table S7: Types of organisms identified using the TAC platform in the specimen collected from all individuals aged 60 and older

Positive targets	# of positive samples	Relative prevalence (%) among specimen positive for 2 targets
Staphylococcus aureus	8	32%
Rhinovirus	6	24%
SARS-CoV-2	3	12%
Human Herpesvirus 4 (EBV)	3	12%
Klebsiella pneumoniae	2	8%
Haemophilus influenzae	1	4%
Human Herpesvirus 5 (CMV)	1	4%

Table S8: Number of positive samples and relative prevalence of organisms identified using the TAC platform among specimens collected from individuals aged 60 and older
